# Efficacy and safety of fremanezumab in patients with migraine and inadequate response to prior preventive treatment: subgroup analyses by country of a randomized, placebo-controlled trial

**DOI:** 10.1186/s10194-021-01232-8

**Published:** 2021-04-16

**Authors:** Egilius L. H. Spierings, Mikko Kärppä, Xiaoping Ning, Joshua M. Cohen, Verena Ramirez Campos, Ronghua Yang, Uwe Reuter

**Affiliations:** 1 Boston Headache Institute, Boston PainCare, 85 1st Ave, Waltham, MA 02451 USA; 2 Research Unit of Clinical Neuroscience, University of Oulu and Medical Research Center, Oulu University Hospital, P.O. Box 8000, Oulu, FI–90014 Finland; 3 Teva Pharmaceutical Industries, Inc., 145 Brandywine Pkwy, West Chester, PA 19380 USA; 4 Department of Neurology, Charité Universitätsmedizin, Charitépl. 1, 10117, Berlin, Germany

**Keywords:** Migraine, Prevention, Fremanezumab, Anti-CGRP, Efficacy, Country

## Abstract

**Background:**

The FOCUS study evaluated the efficacy of migraine preventive medications across different countries within the same patient population, particularly for patients with difficult-to-treat migraine. These prespecified subgroup analyses evaluated efficacy by country in the FOCUS study of fremanezumab in adults with episodic migraine or chronic migraine and documented inadequate response to 2 to 4 migraine preventive medication classes.

**Methods:**

Overall, 838 participants were enrolled in the FOCUS study, a randomized, double-blind, placebo-controlled, parallel-group, phase 3b study performed at 104 sites. For 12 weeks of double-blind treatment, patients were randomized (1:1:1) to quarterly fremanezumab, monthly fremanezumab, or matched placebo. The primary efficacy endpoint was the mean change from baseline in monthly average migraine days over 12 weeks of double-blind treatment, evaluated by country in these subgroup analyses.

**Results:**

Of 14 countries contributing data, the Czech Republic (*n* = 188/838; 22%), the United States (*n* = 120/838; 14%), and Finland (*n* = 85/838; 10%) enrolled the most patients. Changes from baseline in monthly average migraine days over 12 weeks were significantly greater with fremanezumab versus placebo for patients in these countries: Czech Republic (least-squares mean difference versus placebo [95% confidence interval]: quarterly fremanezumab, − 1.9 [− 3.25, − 0.47]; *P* = 0.009; monthly fremanezumab, − 3.0 [− 4.39, − 1.59]; *P* < 0.001), the United States (quarterly fremanezumab, − 3.7 [− 5.77, − 1.58]; *P* < 0.001; monthly fremanezumab, − 4.2 [− 6.23, − 2.13]; *P* < 0.001), and Finland (quarterly fremanezumab, − 3.0 [− 5.32, − 0.63]; *P* = 0.014; monthly fremanezumab, − 3.9 [− 6.27, − 1.44]; *P* = 0.002). Results were comparable for the remaining 9 countries, with the least-squares mean difference versus placebo ranging from – 5.6 to – 2.4 with quarterly fremanezumab and from − 5.3 to − 1.5 with monthly fremanezumab. Incidences of serious adverse events and adverse events leading to discontinuation were low and comparable across countries and treatment groups.

**Conclusions:**

Monthly and quarterly fremanezumab significantly reduced the monthly average number of migraine days versus placebo regardless of country and continent (North America versus Europe) in migraine patients with documented inadequate response to 2 to 4 migraine preventive medication classes.

**Trial registration:**

ClinicalTrials.gov Identifier: NCT03308968.

**Supplementary Information:**

The online version contains supplementary material available at 10.1186/s10194-021-01232-8.

## Background

Migraine affects > 1 billion people worldwide and is associated with functional impairment and substantial economic burden [1–3]. As the leading cause of years lived with disability among individuals < 50 years of age, the burden of migraine and unmet needs are generally higher for patients who have failed ≥ 1 prior preventive treatment [3].

Fremanezumab is a fully humanized monoclonal antibody (IgG isotype 2Δa) that potently and selectively binds to both isoforms of calcitonin gene-related peptide [4]. Fremanezumab has been approved in the United States for the preventive treatment of migraine in adults and in Europe for the prophylaxis of migraine in adults who have ≥ 4 migraine days per month [4, 5]. Fremanezumab has demonstrated efficacy, based on reductions in migraine and headache days, in patients with episodic migraine (EM) and chronic migraine (CM) in the 12-week, randomized, double-blind, placbo-controlled, phase 3 HALO studies [6, 7]. In a subsequent 12-month extension study, that efficacy was maintained over long-term treatment with fremanezumab [8], with no evidence of a wearing-off effect at the end of the quarterly or monthly dosing intervals [9]. In the FOCUS study, fremanezumab treatment significantly reduced migraine and headache days in patients with EM or CM and documented inadequate response to 2 to 4 prior classes of migraine preventive medications [10].

Given the differences in the epidemiology and burden of migraine across different countries [1], there is the potential that the effectiveness of preventive medications for migraine may differ across different countries. Therefore, in a subgroup analysis of the FOCUS study, we evaluated the efficacy of fremanezumab by country in this population of patients with difficult-to-treat migraine. Based on the favorable efficacy results demonstrated in the overall study population, we hypothesized that treatment with quarterly or monthly fremanezumab would be efficacious in patients regardless of their country of residence.

## Methods

This was a preplanned exploratory analysis (for the primary endpoint) of the international, multicenter, randomized, double-blind, placebo-controlled, parallel-group, phase 3b FOCUS study (ClinicalTrials.gov Identifier: NCT03308968) in patients with EM or CM who had documented inadequate response to 2 to 4 prior classes of migraine preventive medications [10]. The FOCUS study was approved by an independent ethics committee or institutional review board at each study site, and each patient provided written informed consent. The study design, eligibility criteria, methods, and statistical analysis methods for that trial have been reported in detail previously [10] and are summarized briefly here.

### Patients

Patients were eligible to participate in the FOCUS study if they were between 18 and 70 years of age, with a diagnosis of migraine (onset ≤ 50 years of age) and a history of migraine for ≥ 12 months prior to screening. Eligible patients also had documented inadequate response within the past 10 years to 2 to 4 migraine preventive medication classes, including angiotensin II receptor blockers (candesartan), anticonvulsants (topiramate), beta blockers (propranolol, metoprolol, atenolol, bisoprolol), calcium channel blockers (flunarizine), onabotulinumtoxinA, tricyclic antidepressants (amitriptyline), or valproic acid. As described previously [10], inadequate response was defined as no clinically meaningful improvement per the treating physician’s judgement after ≥ 3 months of therapy at a stable dose, treatment discontinuation due to adverse events, or the treatment was contraindicated or not suitable for the patient. Acceptable documentation of prior inadequate response included the patient’s medical record (including the name of the medication, duration of treatment, dose, and reason for discontinuing treatment) or an affidavit that provided confirmation of inadequate response [10].

### Study design

The FOCUS study included a screening visit; a 28-day run-in period; a 12-week, double-blind, placebo-controlled treatment period; and a 12-week, open-label treatment period. For the 12-week, double-blind treatment period, patients were randomized (1:1:1) to monthly fremanezumab (Month 1: CM, 675 mg; EM, 225 mg; Months 2 and 3: 225 mg), quarterly fremanezumab (Month 1: 675 mg, Months 2 and 3: placebo), or matched placebo. Efficacy was evaluated using information entered daily by patients in an electronic headache diary throughout the treatment period [10].

### Outcomes

For this analysis, all outcomes were evaluated for subgroups of patients divided by country. The primary efficacy outcome for the FOCUS study was the mean change from baseline in the monthly average number of migraine days over 12 weeks of double-blind treatment. Secondary endpoints, prespecified evaluations, and safety analyses are described within the Methods in Additional file 1.

### Statistical analyses

For the overall FOCUS study population, a sample size of 705 evaluable patients (235 patients per group) completing the study was needed for 90% power to show a 1.8-day difference in monthly migraine days (based on an assumption of a common standard deviation [SD] of 6 days) at an alpha level of 0.05. Assuming a 12% discontinuation rate, 268 patients per treatment group will be randomized in the study. The safety analysis set included all randomized patients who received ≥ 1 dose of study drug. Efficacy analyses were performed on the modified intention-to-treat analysis set, which included all randomized patients who received ≥ 1 dose of study drug and had ≥ 10 days of post-baseline efficacy assessments for the primary endpoint. Efficacy and safety outcomes were summarized using descriptive statistics (ie, sample size, mean, SD, and frequency counts).

For the change from baseline in the monthly average number of migraine days during 12 weeks (primary endpoint of the study), analyses were performed using an analysis of covariance (ANCOVA) method that included treatment, sex, region, special group of treatment failure (patients who had documented inadequate response to valproic acid and documented inadequate response to 2 to 3 other migraine preventive medication classes), migraine classification, and treatment-by-migraine classification interaction as fixed effects and baseline number of migraine days and years since onset of migraine as covariates. A *P* value of < 0.05 was considered significant, and tests are 2-tailed. All summaries and statistical analyses were generated using SAS® software (Version 9.4 of SAS System for Windows, SAS Institute Inc., Cary, NC, USA). Statistical analyses for additional efficacy outcomes are described within the eMethods in the Supplement.

## Results

Overall, 838 patients were randomized in the FOCUS study and received ≥ 1 dose of study drug; 837 patients had ≥ 10 days of post-baseline diary entries for the primary outcome (modified intention-to-treat analysis set). Of the 14 countries contributing data, the Czech Republic, the United States, and Finland were the 3 top-recruiting countries (with each country recruiting ≥ 10% of the total study population; Table S1 in Additional file 2. These 3 countries together recruited 47% (393/838) of patients for the total study (Czech Republic, *n* = 188 [22%]; United States, *n* = 120 [14%]; Finland, *n* = 85 [10%]; Table S1 in Additional file 2). Baseline monthly migraine days were similar across treatment groups within the 3 countries. Overall, baseline monthly migraine days in these 3 top-recruiting countries were similar to those of the total population of the study across all countries (Table S2 in Additional file 3).

**Fig. 1:**
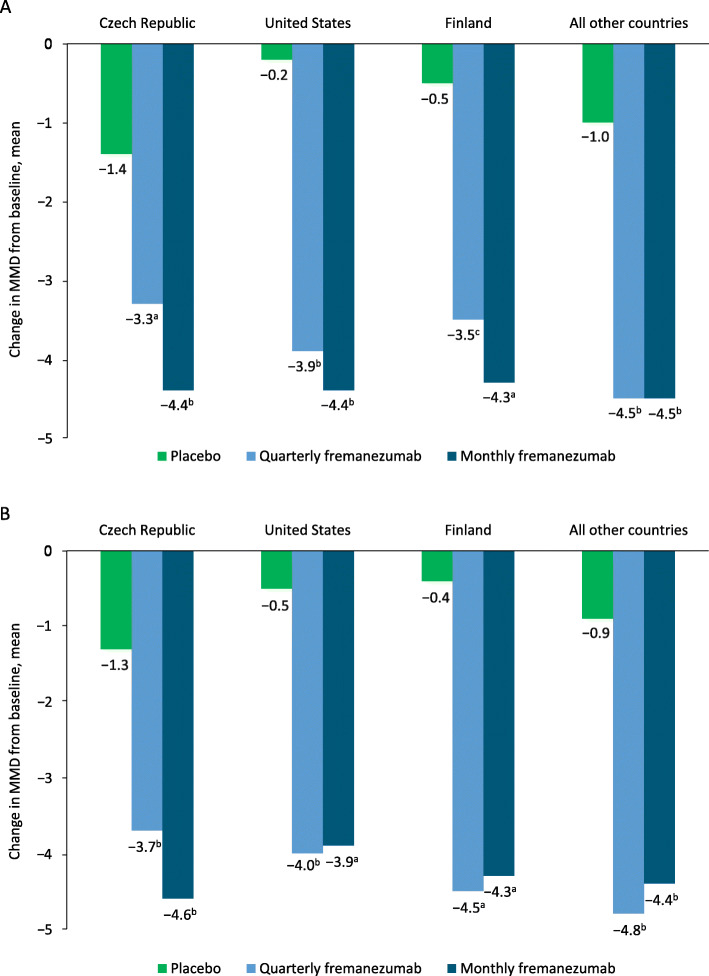
Reductions in MMD from baseline in the 3 top-recruiting countries and all other countries during **a**) 12 weeks and at **b**) 4 weeks. *MMD* monthly migraine days. ^a^*P* < 0.01 versus placebo. ^b^*P* < 0.001 versus placebo. *P* < 0.05 versus placebo

For the primary endpoint, the reduction from baseline in the monthly average number of migraine days during the 12 weeks after the first dose of study drug was significantly greater with both quarterly and monthly fremanezumab versus placebo and was similar across all 3 top-recruiting countries (*P* < 0.05; Table 1, Fig. 1a). Similarly, for all countries excluding the Czech Republic, the United States, and Finland, the least-squares mean (LSM) change from baseline was significantly greater with both fremanezumab dosing regimens versus placebo (*P* < 0.001; Fig. 1a). Among those individual countries, reductions from baseline in the monthly average number of migraine days during the 12 weeks after the first dose of study drug were significantly greater compared with placebo with both dosing regimens of fremanezumab for Germany, Poland, Sweden, Denmark, and the Netherlands; with quarterly fremanezumab for Spain and the United Kingdom; and with monthly fremanezumab for France (*P* < 0.05; Table 1).

**Table 1:** Change From Baseline in the Monthly Average Number of Migraine Days During 12 Weeks of Double-blind Treatment (Primary Efficacy) in Individual Countries and Overall^a^

Country (***n***)	Placebo	Quarterly fremanezumab	Monthly fremanezumab
**All (*n*** **= 838)**	(*n* = 279)	(*n* = 276)	(*n* = 283)
Change from baseline (SE)	− 0.6 (0.34)	− 3.7 (0.34)	− 4.1 (0.34)
Difference from placebo (95% CI)		− 3.1 (− 3.84, − 2.42)	− 3.5 (− 4.19, − 2.78)
*P* value		< 0.001	< 0.001
**Czech Republic (*n*** **= 188)**	(*n* = 60)	(*n* = 65)	(*n* = 63)
Change from baseline (SE)	− 1.4 (0.66)	− 3.3 (0.62)	− 4.4 (0.63)
Difference from placebo (95% CI)		− 1.9 (− 3.25, − 0.47)	− 3.0 (− 4.39, − 1.59)
*P* value		0.009	< 0.001
**USA (*n*** **= 119)**	(*n* = 39)	(*n* = 39)	(*n* = 41)
Change from baseline (SE)	− 0.2 (0.86)	− 3.9 (0.88)	− 4.4 (0.86)
Difference from placebo (95% CI)		− 3.7 (− 5.77, − 1.58)	− 4.2 (− 6.23, − 2.13)
*P* value		< 0.001	< 0.001
**Finland (*n*** **= 85)**	(*n* = 27)	(*n* = 29)	(*n* = 29)
Change from baseline (SE)	− 0.5 (1.14)	− 3.5 (1.04)	− 4.3 (1.14)
Difference from placebo (95% CI)		− 3.0 (− 5.32, − 0.63)	− 3.9 (− 6.27, − 1.44)
*P* value		0.01	0.002
**Belgium (*n*** **= 50)**	(*n* = 15)	(*n* = 18)	(*n* = 17)
Change from baseline (SE)	− 0.4 (1.13)	− 2.8 (1.08)	− 2.8 (1.13)
Difference from placebo (95% CI)		− 2.4 (− 5.41, 0.63)	− 2.4 (− 5.44, 0.67)
*P* value		0.12	0.12
**Denmark (*n*** **= 34)**	(*n* = 12)	(*n* = 11)	(*n* = 11)
Change from baseline (SE)	0 (2.00)	− 4.0 (2.27)	− 3.4 (2.07)
Difference from placebo (95% CI)		− 4.0 (− 7.77, − 0.20)	− 3.4 (− 7.38, 0.53)
*P* value		0.04	0.09
**France (*n*** **= 35)**	(*n* = 13)	(*n* = 10)	(*n* = 12)
Change from baseline (SE)	− 3.1 (1.78)	− 6.0 (1.84)	− 8.4 (1.97)
Difference from placebo (95% CI)		− 2.8 (−7.38, 1.76)	− 5.3 (−9.86, −0.64)
*P* value		0.22	0.03
**Germany (*n*** **= 74)**	(*n* = 25)	(*n* = 24)	(*n* = 25)
Change from baseline (SE)	− 0.5 (1.17)	− 4.7 (1.25)	− 5.2 (1.16)
Difference from placebo (95% CI)		− 4.2 (− 6.76, − 1.61)	− 4.7 (− 7.17, − 2.14)
*P* value		0.002	< 0.001
**The Netherlands (*n*** **= 23)**	(*n* = 7)	(*n* = 8)	(*n* = 8)
Change from baseline (SE)	− 0.1 (1.66)	− 5.6 (1.41)	− 4.6 (1.29)
Difference from placebo (95% CI)		− 5.6 (− 9.52, − 1.60)	− 4.5 (− 8.65, − 0.44)
*P* value		0.009	0.03
**Poland (*n*** **= 66)**	(*n* = 23)	(*n* = 19)	(*n* = 24)
Change from baseline (SE)	− 2.5 (1.13)	− 5.7 (1.35)	− 5.7 (1.12)
Difference from placebo (95% CI)		− 3.1 (− 6.13, − 0.16)	− 3.2 (− 5.77, − 0.59)
*P* value		0.04	0.02
**Spain (*n*** **= 78)**	(*n* = 30)	(*n* = 25)	(*n* = 23)
Change from baseline (SE)	− 0.5 (1.16)	− 3.3 (1.28)	− 2.0 (1.61)
Difference from placebo (95% CI)		− 2.9 (− 5.61, − 0.14)	− 1.5 (− 4.53, 1.51)
*P* value		0.04	0.32
**Sweden (*n*** **= 37)**	(*n* = 12)	(*n* = 12)	(*n* = 13)
Change from baseline (SE)	0.3 (1.85)	− 3.2 (1.91)	− 3.9 (1.72)
Difference from placebo (95% CI)		− 3.5 (− 6.24, − 0.79)	− 4.2 (− 6.94, − 1.46)
*P* value		0.01	0.004
**United Kingdom (*n*** **= 36)**	(*n* = 11)	(*n* = 13)	(*n* = 12)
Change from baseline (SE)	− 1.3 (1.98)	− 6.1 (1.87)	− 4.8 (1.94)
Difference from placebo (95% CI)		− 4.7 (− 8.88, − 0.54)	− 3.5 (− 7.79, 0.82)
*P* value		0.03	0.11

*SE* standard error, *CI* confidence interval, *LSM* least-squares mean

^a^Change from baseline is LSM change

The reduction from baseline in the monthly average number of migraine days at 4 weeks was significantly greater with both quarterly and monthly fremanezumab versus placebo and similar across all top-recruiting countries (*P* < 0.05; Fig. 1b). For patients in the Czech Republic, the LSM change from baseline in monthly average migraine days at 4 weeks was significantly greater versus placebo with quarterly fremanezumab (*P* < 0.001) and monthly fremanezumab (*P* < 0.001). Significantly greater reductions in monthly average migraine days at 4 weeks versus placebo were also observed in patients in the United States with quarterly fremanezumab (*P* < 0.001) and monthly fremanezumab (*P* = 0.001), as well as in patients in Finland (quarterly fremanezumab, *P* = 0.003; monthly fremanezumab, *P* = 0.007).

Reductions from baseline in the monthly average number of headache days of at least moderate severity during 12 weeks of double-blind treatment were significantly greater with both fremanezumab dosing regimens versus placebo in patients in the Czech Republic, the United States, and Finland (*P* ≤ 0.01; Table 2). Reductions from baseline in the monthly average number of headache days of at least moderate severity at 4 weeks of double-blind treatment were also significantly greater with both fremanezumab dosing regimens versus placebo in patients in the Czech Republic, the United States, and Finland (*P* ≤ 0.01; Table 2).

**Table 2:** Effect of Quarterly and Monthly Fremanezumab Doses on Efficacy Responses in the 3 Top-recruiting Countries^a^

	Czech Republic			United States			Finland		
	Placebo (***n*** = 60)	Quarterly fremanezumab (***n*** = 65)	Monthly fremanezumab (***n*** = 63)	Placebo (***n*** = 39)	Quarterly fremanezumab (***n*** = 39)	Monthly fremanezumab (***n*** = 41)	Placebo (***n*** = 27)	Quarterly fremanezumab (***n*** = 29)	Monthly fremanezumab (***n*** = 29)
12 weeks									
Monthly headache days of at least moderate severity									
Change from baseline (SE)	− 0.9 (0.63)	− 3.1 (0.59)	− 4.1 (0.60)	− 0.6 (0.82)	− 3.4 (0.87)	− 5.2 (0.83)	− 0.6 (1.15)	− 3.5 (1.05)	− 4.2 (1.15)
Difference from placebo (95% CI)		− 2.2 (− 3.56, − 0.90)	− 3.2 (− 4.52, − 1.83)		− 2.8 (− 4.86, − 0.76)	− 4.6 (− 6.62, − 2.60)		− 3.0 (− 5.33, − 0.60)	− 3.6 (− 6.06, − 1.17)
*P* value		0.001	< 0.001		0.008	< 0.001		0.01	0.004
Monthly days of any acute headache medication use									
Change from baseline (SE)	− 1.1 (0.65)	− 3.0 (0.61)	− 4.2 (0.62)	− 0.3 (0.78)	− 3.0 (0.81)	− 3.2 (0.78)	− 0.7 (1.17)	− 3.5 (1.07)	− 3.8 (1.18)
Difference from placebo (95% CI)		− 1.9 (− 3.27, − 0.53)	− 3.0 (− 4.41, − 1.64)		− 2.7 (− 4.62, − 0.73)	− 2.9 (− 4.80, − 1.00)		− 2.8 (− 5.23, − 0.40)	− 3.1 (− 5.61, − 0.60)
*P* value		0.007	< 0.001		0.007	0.003		0.02	0.02
4 weeks									
Monthly headache days of at least moderate severity									
Change from baseline (SE)	− 0.6 (0.62)	− 3.3 (0.59)	− 4.1 (0.60)	− 0.5 (0.83)	− 3.3 (0.87)	− 5.1 (0.83)	− 0.6 (1.20)	− 4.7 (1.10)	− 4.7 (1.21)
Difference from placebo (95% CI)		− 2.7 (− 4.04, − 1.36)	− 3.5 (− 4.83, − 2.13)		− 2.8 (− 4.84, − 0.69)	− 4.6 (− 6.61, − 2.55)		− 4.0 (− 6.61, − 1.44)	− 4.1 (− 6.76, − 1.41)
*P* value		< 0.001	< 0.001		0.01	< 0.001		0.003	0.003

*SE* standard error, *CI* confidence interval, *LSM * least-squares mean

^a^Change from baseline is LSM change

**Fig. 2:**
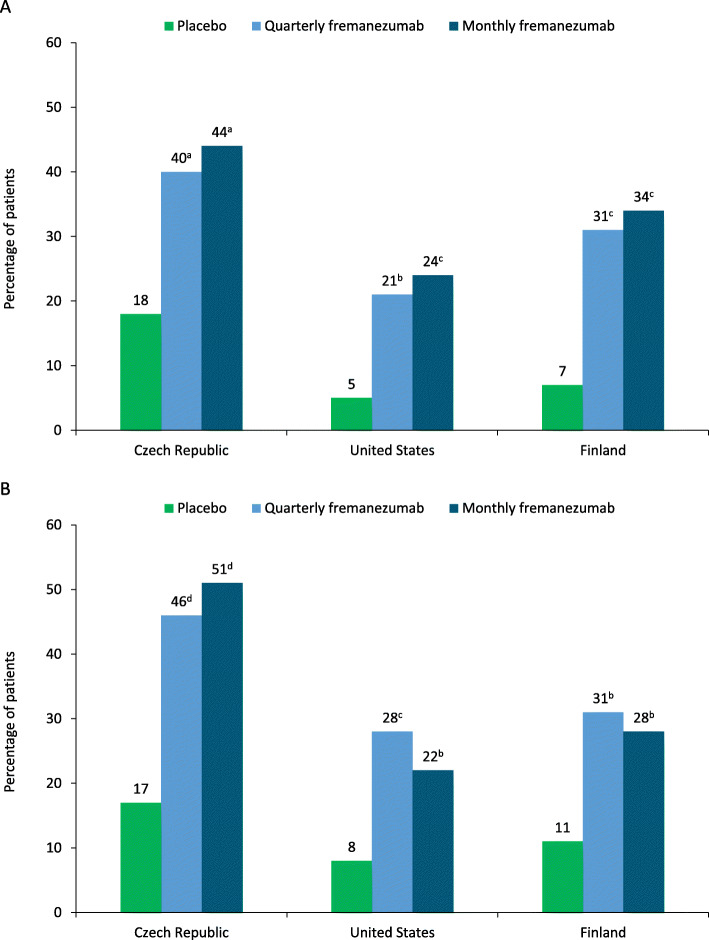
Patients achieving ≥ 50% reduction in MMD during **a**) 12 weeks and at **b**) 4 weeks. *MMD* monthly migraine days. ^a^*P* < 0.01 versus placebo. ^b^*P* > 0.05 versus placebo. ^c^*P* < 0.05 versus placebo. ^d^*P* ≤ 0.001 versus placebo

The proportion of patients who achieved ≥ 50% reduction in the monthly average number of migraine days during 12 weeks of double-blind treatment was significantly higher with quarterly and monthly fremanezumab versus placebo in patients in the Czech Republic and Finland, and with monthly fremanezumab versus placebo in patients in the United States (Fig. 2). In patients in the Czech Republic, the proportion of patients who achieved ≥ 50% reduction in monthly average migraine days was significantly higher versus placebo with quarterly fremanezumab (odds ratio [OR] versus placebo [95% confidence interval (CI)], 3.11 [1.35, 7.13]; *P* = 0.008), and monthly fremanezumab (3.77 [1.64, 8.67]; *P* = 0.002). The proportion of patients who achieved ≥ 50% reduction in monthly average migraine days was also significantly higher versus placebo with monthly fremanezumab in the United States (quarterly fremanezumab, OR [95% CI], 5.11 [0.95, 27.40]; *P* = 0.057; monthly fremanezumab, 6.25 [1.20, 32.57]; *P* = 0.030) and with both dosing regimens in Finland (quarterly fremanezumab, 6.57 [1.24, 34.77]; *P* = 0.027; monthly fremanezumab, 7.16 [1.38, 37.23]; *P* = 0.019).

The proportion of patients who achieved ≥ 50% reduction in the monthly average number of migraine days at 4 weeks of double-blind treatment was higher with quarterly and monthly fremanezumab versus placebo in patients in the United States, and Finland and significantly higher in patients in the Czech Republic (Fig. 2). The proportions of patients who achieved ≥50% reduction in monthly migraine days at 4 weeks was significantly higher versus placebo with both fremanezumab dosing regimens in the Czech Republic (quarterly fremanezumab, OR [95% CI], 4.51 [1.94, 10.51]; *P* < 0.001; monthly fremanezumab, 5.46 [2.33, 12.76]; *P* < 0.001) and with quarterly fremanezumab in the United States (quarterly fremanezumab, 5.48 [1.28, 23.45]; *P* = 0.022; monthly fremanezumab, 3.49 [0.81, 15.07]; *P* = 0.094). Differences between the fremanezumab and placebo groups did not reach statistical significance in Finland (quarterly fremanezumab, OR [95% CI], 4.19 [0.97, 18.10]; *P* = 0.055; monthly fremanezumab, 3.25 [0.75, 14.12]; *P* = 0.116).

**Fig. 3:**
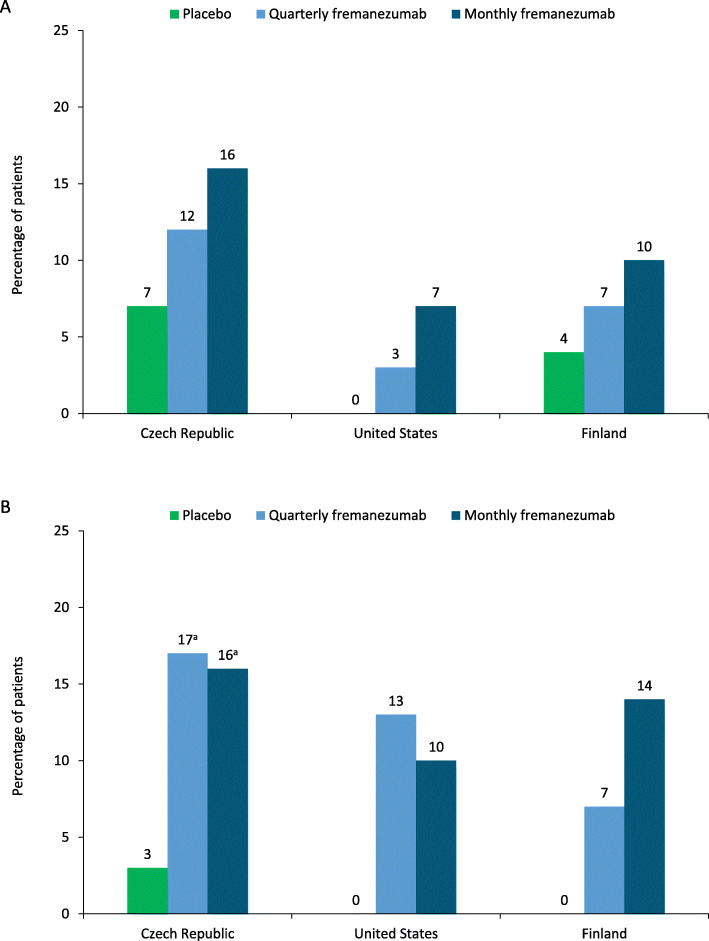
Patients achieving ≥ 75% reduction in MMD during **a**) 12 weeks and at **b**) 4 weeks. *MMD* monthly migraine days. ^a^*P* < 0.05 versus placebo

The proportion of patients who achieved ≥ 75% reduction in the monthly average number of migraine days during 12 weeks of double-blind treatment was higher with quarterly and monthly fremanezumab versus placebo in patients in the Czech Republic, the United States, and Finland (Fig. 3). The proportion of patients who achieved ≥ 75% reduction in the monthly average number of migraine days at 4 weeks of double-blind treatment was higher with quarterly and monthly fremanezumab versus placebo in patients in the Czech Republic, the United States, and Finland (Fig. 3).

Reductions from baseline in the monthly average number of days of any acute headache medication use during the 12 weeks after the first dose of study drug were significantly greater with both quarterly and monthly fremanezumab versus placebo in patients in the Czech Republic, the United States, and Finland (*P* ≤ 0.02; Table 2). Placebo-adjusted reductions in monthly days of any acute headache medication use were approximately 2 to 3 days for patients in the Czech Republic and 3 days for patients in the United States and Finland.

Improvements from baseline in headache-related disability, as measured by reductions in 6-item Headache Impact Test (HIT-6) scores, were higher with both fremanezumab dosing regimens versus placebo, although not all differences reached statistical significance (Table S3 in Additional file 4). Reductions in HIT-6 scores versus placebo of approximately 2 to 3 points were observed for patients in the Czech Republic, 2 to 4.5 points for those in the United States, and 4.5 to 5 points for those in Finland.

Improvements in migraine-related disability, as measured by reductions in Migraine Disability Assessment (MIDAS) scores from baseline, were generally higher with fremanezumab versus placebo; however, these between-group differences did not reach statistical significance for all comparisons (Table S3 in Additional file 4). Reductions in MIDAS scores versus placebo ranged from approximately 2 to 6.5 points for patients in the Czech Republic, 6 to 12 points for those in the United States, and 20 to 24 points in for those in Finland.

The proportion of responders on the Patient Global Impression of Change (PGIC) scale was higher with both quarterly and monthly fremanezumab versus placebo in patients in the Czech Republic, the United States, and Finland, although all differences did not reach statistical significance (Table S3 in Additional file 4). Approximately 50% to 80% of patients receiving fremanezumab across these three countries were considered PGIC responders (ie, reported improvements ranging from moderately better to a great deal better), except for patients receiving quarterly fremanezumab in the United States (31%).

Incidences of injection-site reaction adverse events, other adverse events, serious adverse events, and adverse events leading to discontinuation were similar across treatment groups in the 3 top-recruiting countries (Table 3). No severe hypersensitivity reactions or cases of anaphylaxis were reported. The most common adverse events across all treatment groups in Europe and the United States were injection-site erythema, injection-site induration, and injection-site pain (Table S4 in Additional file 5). No deaths were reported. In the 3 top-recruiting countries, serious adverse events were reported for 3 (2%) of 126 patients who received placebo, 1 (< 1%) of 134 who received quarterly fremanezumab, and none of 133 who received monthly fremanezumab. No safety signals were identified in this subgroup analysis.

**Table 3:** Tolerability in the 3 Top-recruiting Countries (Czech Republic, United States, and Finland)

AEs, no. (%)	Placebo (***n*** = 126)	Monthly fremanezumab (***n*** = 134)	Quarterly fremanezumab (***n*** = 133)
≥ 1 injection-site reaction AE	15 (12)	21 (16)	14 (10)
≥ 1 AE	52 (41)	63 (47)	55 (41)
≥ 1 SAE	3 (2)	0	1 (< 1)
AEs leading to discontinuation	2 (2)	0	2 (1)
AEs^a^			
Injection-site erythema	6 (5)	9 (7)	6 (4)
Injection-site pain	4 (3)	9 (7)	3 (2)
Injection-site induration	3 (2)	4 (3)	7 (5)
Injection-site bruising	2 (2)	2 (2)	2 (1)
Injection-site pruritus	2 (2)	2 (2)	2 (1)
Nasopharyngitis	6 (5)	6 (5)	4 (3)
Upper respiratory tract infection	1 (< 1)	–	7 (5)
Influenza	0	2 (2)	4 (3)
Nausea	2 (2)	3 (2)	2 (1)
Constipation	2 (2)	3 (2)	0
Dyspepsia	2 (2)	0	2 (1)
Urinary tract infection	2 (2)	1 (< 1)	0
Foot fracture	0	2 (2)	0
Road traffic accident	0	2 (2)	0
International normalized ratio increased	2 (2)	7 (5)	2 (1)
Hemoglobin decreased	0	2 (2)	0
Neck pain	0	1 (< 1)	3 (2)
Pain in extremity	2 (2)	0	3 (2)
Insomnia	1 (< 1)	3 (2)	3 (2)
Anxiety	0	1 (< 1)	2 (1)
Alopecia	2 (2)	1 (< 1)	1 (< 1)

*AE*, adverse event, *SAE*, serious adverse event

^a^AEs that occurred in ≥ 2 patients in any treatment group were reported

## Discussion

The primary efficacy analysis demonstrated statistical significance in favor of fremanezumab in the 3 top-recruiting countries, the Czech Republic, the United States, and Finland. As compared with placebo, fremanezumab treatment resulted in approximately 2- to 3-day reductions from baseline in monthly migraine days over 12 weeks of treatment (primary endpoint) in the Czech Republic and approximately 3.5- to 4-day reductions in the United States and Finland. Similarly, a reduction of approximately 3.5 days was found for this endpoint for all countries excluding these 3 top-recruiting countries, with reductions ranging from approximately 2 to 8 days across those countries. Fremanezumab also reduced monthly headache days of at least moderate severity by 2.5 to 4.5 days as early as 4 weeks after starting treatment across the 3 top-recruiting countries, with similar results observed during the 12 weeks after the first dose of study drug. For patients in these 3 top-recruiting countries, the observed reductions in days with any acute headache medication use of approximately 2 to 3 days were also similar to the results reported previously for the overall FOCUS population [10]. The consistency among the patient subgroups suggests that these results are generalizable and not limited to a predominant recruiting country.

Across different countries, the epidemiology and burden of migraine vary [1], along with the severity of headache pain and migraine-related disability [11]. The use of acute and preventive prescription medications for migraine also differs by country [12]. Furthermore, there is the potential for differences in clinical trial execution in different regions. For example, the prevalence of migraine is higher in Finland than in the Czech Republic or the United States, as are the number of migraine-associated years lived with disability [1, 13]. Although each clinical site participating in a clinical trial is expected to closely follow clinical trial protocols and guidelines, the heterogeneity of resource availability, infrastructure, clinical trial regulatory requirements, sociocultural practices, and patient education and support among countries may result in differences in clinical trial execution [14, 15].

The results of the FOCUS study provide evidence for the efficacy of fremanezumab in a broad population of patients with difficult-to-treat EM or CM and prior inadequate response to 2 to 4 prior migraine preventive medication classes [10]. The results of the current subgroup analysis, which showed consistent efficacy across countries in this population with inadequate response to multiple prior preventive treatments, are of note given the potential differences in the burden of migraine and the severity of associated headache and disability between countries [1, 11]. Although in the current analyses, there were no substantial differences in efficacy across the different countries involved in the clinical trial, the impact of geographic composition on the outcomes of international studies may be an area of future research to determine how to best manage potential differences in trial execution across sites in different countries.

For patients enrolled from the United States, the odds of achieving a ≥ 50% reduction in monthly migraine days were approximately 5 to 6 times higher with fremanezumab than with placebo during the 12 weeks after the first dose of study drug. For the same endpoint, the odds were approximately 3 to 3.5 times higher in the Czech Republic and 6.5 to 7 times higher in Finland.

Patient-reported outcomes were also favorable for fremanezumab as compared to placebo in the current analysis. Both quarterly and monthly fremanezumab improved headache-related disability, with decreases in HIT-6 scores of 2 to 5 points more than with placebo and MIDAS scores of up to 24 points more than with placebo across the 3 top-recruiting countries. According to the recent American Headache Society consensus statement [16], a ≥ 5-point reduction in HIT-6 scores was considered a clinically meaningful response to migraine therapy. In addition, a ≥ 5-point reduction in MIDAS scores for patients with baseline scores of 11 to 20 or a 30% reduction in MIDAS scores for patients with baseline scores > 20 is indicative of clinically meaningful improvements in migraine-related disability.

Fremanezumab was well tolerated. No safety signals were identified, and the incidence of injection-site reaction adverse events, other adverse events, and serious adverse events was similar to that of placebo. Only 2 patients (1%) in the top-recruiting countries across both fremanezumab dosing regimens had an adverse event leading to discontinuation, which was the same amount as in the placebo group. Injection-site reactions were the most common type of adverse events. Across the current subgroups and the FOCUS study population, no deaths, cases of anaphylaxis, or severe hypersensitivity reactions related to fremanezumab treatment have been reported [10].

The results reported here may be subject to certain limitations. This analysis included relatively small numbers of patients in each country-based subgroup, and the subgroups differed in size, making comparisons across countries challenging. Further, recruitment was relatively low in some larger countries, including the United Kingdom, Italy, and Switzerland, which led to limited power to evaluate between-group differences in efficacy for the primary endpoint. Results were not analyzed separately for those countries (Italy and Switzerland) with extremely low sample numbers (< 10 patients). In addition, patients from the Czech Republic more commonly had EM and a lower number of prior preventive medications with inadequate response than those in the United States or Finland, suggesting less severe disease. Additionally, while the country subgroups and primary endpoint were prespecified, analysis of the secondary endpoints was performed post hoc. Since the data for all outcome measures were captured prospectively, the impact of this limitation is likely minimal.

## Conclusion

This subgroup analysis of the FOCUS study is the first to evaluate the efficacy of a migraine preventive medication across individual countries. The consistency of response across the high-recruiting countries and the overall group of countries excluding these 3 top recruiters suggests that the correct population was selected for the overall study and is representative of a population with difficult-to-treat migraine and inadequate response to up to 4 migraine preventive medication classes. Since efficacy results were similar across these 3 top-recruiting countries and in line with the total population, these findings further demonstrate that fremanezumab is consistently effective across different migraine patient populations, including patients with difficult-to-treat migraine, across the United States and Europe.

## Supplementary Information

**Additional file 1: Supplemental Methods.** Description of outcomes and statistical analyses for secondary endpoints, prespecified evaluations, and safety analyses.

**Additional file 2.** Enrollment Across Countries in the FOCUS Study. Number and proportion of patients enrolled in the FOCUS study from each country overall and by treatment group.

**Additional file 3.** Mean (SD) Monthly Migraine Days and Headache Days of at Least Moderate Severity at Baseline.

**Additional file 4.** Change in Patient-reported Outcomes 4 Weeks After the Third Dose of Study Drug. Change in 6-item Headache Impact Test (HIT-6) and Migraine Disability Assessment (MIDAS) scores and Patient Global Impression of Change (PGIC) responder rates from baseline to 4 weeks after the third dose of study drug. PGIC responder was defined as a patient who reported a rating of 5 to 7 (moderately better, better, or a great deal better) on the PGIC.

**Additional file 5.** Safety in Patients From Europe and the United States. Injection-site adverse events in patients from Europe and the United States by treatment group.

## Data Availability

Anonymized data, as described in this manuscript, will be shared upon request from any qualified investigator by the author investigators or Teva Branded Pharmaceutical Products R&D, Inc.

## Abbreviations

ANCOVA: Analysis of covariance
CI: Confidence interval
CM: Chronic migraine
EM: Episodic migraine
HIT-6: 6-item Headache Impact Test
LSM: Least-squares mean
MIDAS: Migraine Disability Assessment
OR: Odds ratio
PGIC: Patient Global Impression of Change
