# Supplementary material for: Efficacy and safety of fremanezumab in patients with migraine and inadequate response to prior preventive treatment: subgroup analyses by country of a randomized, placebo-controlled trial
Source: J Headache Pain. 2021 Apr 16;22(1):26. doi: 10.1186/s10194-021-01232-8 (PMC8052719; doi:10.1186/s10194-021-01232-8)
Supplement: Supplementary file 1 — Additional file 1: Supplemental Methods. Description of outcomes and statistical analyses for secondary endpoints, prespecified evaluations, and safety analyses. [file 10194_2021_1232_MOESM1_ESM.docx]

**Additional file 1: Supplemental Methods**

**Outcomes**

Secondary endpoints included the proportion of patients achieving ≥50% reduction in the monthly average number of migraine days, the mean change from baseline in monthly average headache days of at least moderate severity, and the mean change from baseline in average days of any acute headache medication use during the 12-week period after the first dose of study drug. The mean change from baseline in the monthly average number of migraine days, the proportion of patients achieving ≥50% reduction in the monthly average number of migraine days, and the mean change from baseline in average headache days of at least moderate severity were also evaluated during the 4-week period after the first dose of study drug.

Prespecified evaluations included the proportion of patients achieving ≥75% reduction in the monthly average number of migraine days during the 4- and 12-week periods after the first dose of study drug and the mean change from baseline in the 6-item Headache Impact Test (HIT-6), Migraine Disability Assessment (MIDAS), and Patient Global Impression of Change (PGIC) scores at 4 weeks after the third dose of study drug.

Tolerability was evaluated based on adverse event reporting. Adverse events were coded using the *Medical Dictionary for Regulatory Activities* (MedDRA) version 18.1.

**Statistical Analyses**

Changes from baseline during 12 weeks of double-blind treatment in other continuous efficacy outcomes (ie, headache days of at least moderate severity, days of use of acute headache medication) were analyzed similarly to the primary endpoint. Changes from baseline over the first 4 weeks of treatment in the monthly average number of migraine days, headache days of at least moderate severity, and days with acute headache medication use were evaluated using a mixed-effects repeated measures (MMRM) analysis method, which included treatment, sex, region, special group of treatment failure, migraine classification, month, treatment-by-migraine classification interaction, treatment-by-month interaction, and treatment-by-migraine classification-by-month interaction as fixed effects and baseline value and years since onset of migraine as covariates.

For the proportion of patients achieving a ≥50% and ≥75% reduction in the monthly average number of migraine days, a logistic regression model was used with stratification factors (as randomized) and the following effects: treatment, sex, region, special group of treatment failure (yes or no), and migraine classification (EM or CM). Study participants who discontinued early were considered non-responders for this analysis. A PGIC responder was defined as a patient who reported a rating of 5–7 (moderately better, better, or a great deal better) on the PGIC.
