# Supplementary material for: Efficacy and safety of fremanezumab in patients with migraine and inadequate response to prior preventive treatment: subgroup analyses by country of a randomized, placebo-controlled trial
Source: J Headache Pain. 2021 Apr 16;22(1):26. doi: 10.1186/s10194-021-01232-8 (PMC8052719; doi:10.1186/s10194-021-01232-8)
Supplement: Supplementary file 2 — Additional file 2. Enrollment Across Countries in the FOCUS Study. Number and proportion of patients enrolled in the FOCUS study from each country overall and by treatment group. [file 10194_2021_1232_MOESM2_ESM.docx]

**Additional file 2: Table S1. Enrollment Across Countries in the FOCUS Study**

| **Country, No. (%)^a^** | **Placebo**  **(n=279)** | **Quarterly Fremanezumab**  **(n=276)** | **Monthly Fremanezumab**  **(n=283)** | **Total**  **(N=838)** |
| --- | --- | --- | --- | --- |
| Czech Republic | 60 (22) | 65 (24) | 63 (22) | 188 (22) |
| United States | 40 (14) | 39 (14) | 41 (14) | 120 (14) |
| Finland | 27 (10) | 29 (11) | 29 (10) | 85 (10) |
| Spain | 30 (11) | 25 (9) | 23 (8) | 78 (9) |
| Germany | 25 (9) | 24 (9) | 25 (9) | 74 (9) |
| Poland | 23 (8) | 19 (7) | 24 (8) | 66 (8) |
| Belgium | 15 (5) | 18 (7) | 17 (6) | 50 (6) |
| Sweden | 12 (4) | 12 (4) | 13 (5) | 37 (4) |
| United Kingdom | 11 (4) | 13 (5) | 12 (4) | 36 (4) |
| France | 13 (5) | 10 (4) | 12 (4) | 35 (4) |
| Denmark | 12 (4) | 11 (4) | 11 (4) | 34 (4) |
| Netherlands | 7 (3) | 8 (3) | 8 (3) | 23 (3) |
| Italy | 3 (1) | 3 (1) | 4 (1) | 10 (1) |
| Switzerland | 1 (<1) | 0 | 1 (<1) | 2 (<1) |

^a^Percentage is of total population in corresponding treatment group.
