# Supplementary material for: Efficacy and safety of fremanezumab in patients with migraine and inadequate response to prior preventive treatment: subgroup analyses by country of a randomized, placebo-controlled trial
Source: J Headache Pain. 2021 Apr 16;22(1):26. doi: 10.1186/s10194-021-01232-8 (PMC8052719; doi:10.1186/s10194-021-01232-8)
Supplement: Supplementary file 3 — Additional file 3. Mean (SD) Monthly Migraine Days and Headache Days of at Least Moderate Severity at Baseline. [file 10194_2021_1232_MOESM3_ESM.docx]

| **Country** | **Placebo**  **(n=147)** | **Quarterly Fremanezumab**  **(n=159)** | **Monthly Fremanezumab**  **(n=155)** |
| --- | --- | --- | --- |
| **Czech Republic** | (n=60) | (n=65) | (n=63) |
| Baseline monthly migraine days | 12.3 (5.67) | 11.9 (5.28) | 12.1 (5.12) |
| Baseline headache days of at least moderate severity | 10.7 (4.82) | 10.5 (5.39) | 10.7 (5.20) |
| **United States** | (n=39) | (n=39) | (n=41) |
| Baseline monthly migraine days | 15.5 (6.73) | 16.4 (7.23) | 15.7 (6.38) |
| Baseline headache days of at least moderate severity | 14.2 (6.80) | 13.5 (7.72) | 14.7 (6.55) |
| **Finland** | (n=27) | (n=29) | (n=29) |
| Baseline monthly migraine days | 14.7 (5.15) | 15.2 (6.20) | 14.3 (5.02) |
| Baseline headache days of at least moderate severity | 13.6 (5.30) | 13.8 (6.90) | 12.9 (5.87) |
| **All countries** | (n=279) | (n=276) | (n=283) |
| Baseline monthly migraine days | 14.3 (6.12) | 14.1 (5.61) | 14.1 (5.58) |
| Baseline headache days of at least moderate severity | 12.8 (5.92) | 12.4 (5.84) | 12.7 (5.82) |

**Additional file 3: Table S2. Mean (SD) Monthly Migraine Days and Headache Days of at Least Moderate Severity at Baseline**

SD, standard deviation.
