# Supplementary material for: Efficacy and safety of fremanezumab in patients with migraine and inadequate response to prior preventive treatment: subgroup analyses by country of a randomized, placebo-controlled trial
Source: J Headache Pain. 2021 Apr 16;22(1):26. doi: 10.1186/s10194-021-01232-8 (PMC8052719; doi:10.1186/s10194-021-01232-8)
Supplement: Supplementary file 4 — Additional file 4. Change in Patient-reported Outcomes 4 Weeks After the Third Dose of Study Drug. Change in 6-item Headache Impact Test (HIT-6) and Migraine Disability Assessment (MIDAS) scores and Patient Global Impression of Change (PGIC) responder rates from baseline to 4 weeks after the third dose of study drug. PGIC responder was defined as a patient who reported a rating of 5 to 7 (moderately better, better, or a great deal better) on the PGIC. [file 10194_2021_1232_MOESM4_ESM.docx]

**Additional file 4: Table S3. Change in Patient-reported Outcomes 4 Weeks After the Third Dose of Study Drug**

|  | **Czech Republic** | | | **United States** | | | **Finland** | | |
| --- | --- | --- | --- | --- | --- | --- | --- | --- | --- |
|  | **Placebo**  **(n=60)** | **Quarterly Fremanezumab**  **(n=65)** | **Monthly Fremanezumab**  **(n=63)** | **Placebo**  **(n=39)** | **Quarterly Fremanezumab**  **(n=39)** | **Monthly Fremanezumab**  **(n=41)** | **Placebo**  **(n=27)** | **Quarterly Fremanezumab**  **(n=29)** | **Monthly Fremanezumab**  **(n=29)** |
| 4 weeks after third dose | | | | | | | | | |
| HIT-6 score | | | | | | | | | |
| Change from baseline (SE) | –3.5 (1.25) | –5.8 (1.17) | –6.7 (1.20) | –1.9 (1.19) | –3.9 (1.31) | –6.5 (1.15) | –0.4 (1.46) | –4.8 (1.37) | –5.7 (1.46) |
| Difference from placebo (95% CI) |  | –2.2 (–4.89, 0.39) | –3.2 (–5.90, –0.50) |  | –2.0 (–4.96, –0.94) | –4.6 (–7.38, –1.88) |  | –4.5 (–7.49, –1.45) | –5.3 (–8.40, –2.21) |
| *P* value |  | 0.10 | 0.02 |  | 0.18 | 0.001 |  | 0.004 | 0.001 |
| MIDAS score | | | | | | | | | |
| Change from baseline (SE) | –19.0 (4.77) | –21.0 (4.45) | –25.5 (4.58) | –9.4 (8.26) | –15.6 (9.09) | –21.9 (8.04) | –9.3 (10.40) | –33.4 (9.82) | –29.4 (10.28) |
| Difference from placebo (95% CI) |  | –2.0 (–12.07, 8.10) | –6.5 (–16.76, 3.75) |  | –6.1 (–26.73, 14.46) | –12.4 (–31.64, 6.75) |  | –24.0 (–45.59, –2.45) | –20.1 (–42.05, 1.88) |
| *P* value |  | 0.70 | 0.21 |  | 0.56 | 0.20 |  | 0.02 | 0.07 |
| PGIC^a^ | | | | | | | | | |
| Responders, No. (%) | 26 (43) | 38 (58) | 49 (78) | 10 (26) | 12 (31) | 23 (56) | 3 (11) | 18 (62) | 14 (48) |
| *P* value |  | 0.10 | <0.001 |  | 0.50 | 0.007 |  | <0.001 | 0.003 |

HIT-6, 6-item Headache Impact Test; SE, standard error; CI, confidence interval; MIDAS, Migraine Disability Assessment; PGIC, Patient Global Impression of Change.

^a^PGIC responder was defined as a patient who reported a rating of 5 to 7 (moderately better, better, or a great deal better) on the PGIC.
