# Supplementary material for: Efficacy and safety of fremanezumab in patients with migraine and inadequate response to prior preventive treatment: subgroup analyses by country of a randomized, placebo-controlled trial
Source: J Headache Pain. 2021 Apr 16;22(1):26. doi: 10.1186/s10194-021-01232-8 (PMC8052719; doi:10.1186/s10194-021-01232-8)
Supplement: Supplementary file 5 — Additional file 5. Safety in Patients From Europe and the United States. Injection-site adverse events in patients from Europe and the United States by treatment group. [file 10194_2021_1232_MOESM5_ESM.docx]

**Additional file 5: Table S4. Safety in Patients From Europe and the United States.**

|  | **Europe** | | | **United States^a^** | | |
| --- | --- | --- | --- | --- | --- | --- |
| **AEs, No. (%)** | **Placebo**  **(n=238)** | **Quarterly Fremanezumab**  **(n=237)** | **Monthly Fremanezumab**  **(n=243)** | **Placebo**  **(n=39)** | **Quarterly Fremanezumab**  **(n=42)** | **Monthly Fremanezumab**  **(n=39)** |
| ≥1 injection-site reaction AE | 23 (10) | 25 (11) | 26 (11) | 10 (26) | 10 (26) | 8 (19) |
| AEs | | | | | | |
| Injection-site erythema | 10 (4) | 17 (7) | 13 (5) | 5 (13) | 2 (5) | 3 (7) |
| Injection-site induration | 9 (4) | 8 (3) | 7 (3) | 3 (8) | 4 (10) | 6 (14) |
| Injection-site pain | 5 (2) | 5 (2) | 6 (2) | 3 (8) | 6 (15) | 3 (7) |
| Injection-site bruising | 1 (<1) | 2 (<1) | 5 (2) | 1 (3) | 0 | 0 |
| Injection-site paresthesia | 3 (1) | 4 (2) | 3 (1) | – | – | – |
| Injection-site pruritus | 2 (<1) | 2 (<1) | 3 (1) | – | – | – |
| Injection-site rash | 2 (<1) | 2 (<1) | 3 (1) | – | – | – |
| Injection-site warmth | 0 | 1 (<1) | 3 (1) | – | – | – |
| Injection-site discoloration | 1 (<1) | 2 (<1) | 1 (<1) | – | – | – |
| Injection-site hematoma | 0 | 2 (<1) | 0 | – | – | – |

AE, adverse event.

^a^The following safety data was not collected for patients in the United States: injection-site paresthesia, injection-site pruritus, injection-site rash, injection-site warmth, injection-site discoloration, and injection-site hematoma.
